# Association Between Enhanced Hydration Therapy and Improved Prognosis in Patients With Acute Ischemic Stroke Who Were Treated With Thrombolytics: A Preliminary Study

**DOI:** 10.1155/emmi/1220736

**Published:** 2025-04-22

**Authors:** Leng Chieh Lin, Chen-June Seak, Yen Chu Huang, Yuan Hsiung Tsai, Jen Tsung Yang, Kai-Hsiang Wu, Chia-Peng Chang, Yen Yun Tsai

**Affiliations:** ^1^Department of Emergency Medicine, Chang Gung Memorial Hospital, No. 8, Sec. W., Jiapu Rd., Puzi, Chiayi 613, Taiwan; ^2^Shu-Zen Junior College of Medicine and Management, No. 452, Huanqiu Rd., Luzhu, Kaohsiung 821, Taiwan; ^3^Department of Emergency Medicine, Chang Gung Memorial Hospital, No. 5, Fuxing St., Guishan, Taoyuan 333, Taiwan; ^4^Medical Center and College of Medicine, Chang Gung University, No. 259, Wenhua 1st Rd., Guishan, Taoyuan 333, Taiwan; ^5^Department of Emergency Medicine, New Taipei Municipal Tucheng Hospital, No. 6, Sec. 2, Jincheng Rd., Tucheng, New Taipei 236, Taiwan; ^6^Department of Neurology, Chang Gung Memorial Hospital, No. 8, Sec. W., Jiapu Rd., Puzi, Chiayi 613, Taiwan; ^7^Department of Diagnostic Radiology, Chang Gung Memorial Hospital, No. 8, Sec. W., Jiapu Rd., Puzi, Chiayi 613, Taiwan; ^8^Division of Neurosurgery, Department of Surgery, Chang Gung Memorial Hospital, Chang Gung University College of Medicine, No. 2, Sec. W., Jiapu Rd., Puzi, Chiayi 613, Taiwan; ^9^Department of Nursing, Chang Gung University of Science and Technology, Chiayi Campus, Chiayi, Taiwan; ^10^Graduate Institute of Clinical Medical Sciences, College of Medicine, Chang Gung University, Taoyuan, Taiwan; ^11^Department of Dermatology, Changhua Christian Hospital, Changhua, Taiwan

**Keywords:** BUN/Cr ratio, hypovolemia, ischemic stroke, prognosis, thrombolytic therapy

## Abstract

**Introduction:** Hypovolemia affects the clinical outcomes and efficacy of thrombolytic therapies such as recombinant tissue plasminogen activator (rt-PA). Hence, it plays an essential role in stroke management. Blood urea nitrogen-to-creatinine ratio (BCR) is an indicator of hypovolemia and is a promising area of further investigation.

**Methods:** This study assessed the efficacy of enhanced hydration therapy in patients with acute ischemic stroke (AIS) who had an elevated BCR and were receiving rt-PA treatment. The outcomes between patients with AIS who received enhanced hydration therapy (the study group) and those with AIS who received standard hydration therapy (the historical control group) were compared. Eligible patients received 0.9% NaCl intravenous infusion at a volume of 20 mL/kg body weight. Then, a bolus injection of one-third of the total volume was administered, and the remaining two-third was continuously infused over 8 h. Next, a maintenance infusion of 40–80 mL/h was administered within 16 h. The primary outcomes were 3-month functional recovery and early neurological deterioration.

**Results:** This analysis included 20 patients with AIS and 170 historical controls. The study and historical control groups did not significantly differ in terms of demographic characteristics, baseline stroke severity, and biochemical parameters. However, the study group had a higher prevalence of hypertension than the historical control group. Further, the study group had significantly better 3-month functional outcomes than the historical control group (*p*=0.018). In particular, 45.0% of patients in the study group and 21.2% in the control group achieved a modified Rankin Scale score of ≤ 2. Based on a subgroup analysis, patients with a lower stroke severity (National Institutes of Health Stroke Scale [NIHSS] scores of 4–10) in the study group exhibited significant improvements in functional outcomes. Meanwhile, patients with a higher stroke severity (NIHSS scores of 11–24) did not present with comparable benefits. The high stroke severity group had a higher complication rate than the low stroke severity group. However, the results did not significantly differ. Importantly, none of the patients who received enhanced hydration therapy developed adverse events.

**Conclusion:** Enhanced hydration therapy can improve outcomes in patients with stroke who had an elevated BCR and who received rt-PA treatment. Further, it is not associated with significant complications.

## 1. Introduction

Hypovolemia is a significant concern in stroke management, affecting 29%–45% of patients admitted due to acute ischemic stroke (AIS) [1]. Variations in the prevalence of hypovolemia are attributed to differences in definitions, diagnostic methods, and the timing of measuring markers such as blood urea nitrogen-to-creatinine ratio (BCR) [2, 3]. The risk factors of hypovolemia in patients with stroke who are hospitalized include advanced age, female sex, total anterior circulation syndrome, use of diuretics [4, 5], and dysphagia [2]. In patients with AIS, hypovolemia can exacerbate cerebral infarction [5–7]. Moreover, it is an independent predictor of early clinical deterioration within the first 3 days after stroke [8–11]. Therefore, it can contribute to poor outcomes.

An elevated BCR, urine specific gravity, and plasma osmolality are some of the commonly used indicators in assessing hypovolemia in patients with AIS [12]. BCR is a practical and widely accessible marker of hydration status [4, 13]. A high BCR is caused by various factors such as hypovolemia, catabolic status, gastrointestinal bleeding, and side effects of medications [14]. Previous studies have revealed that an elevated BCR is associated with hypovolemia in 60%–70% of cases [15, 16]. In addition, it is related to thromboembolism. Meanwhile, hypovolemia negatively affects the long-term prognosis of patients with AIS who received thrombolytics [17–19]. However, the interaction between hypovolemia and thrombolytic therapies, such as recombinant tissue plasminogen activator (rt-PA), is significantly underexplored.

The significance of hypovolemia in AIS, particularly in terms of patient outcomes and responses to thrombolytic therapy, is increasingly recognized in the literature [4, 19]. BCR consistently emerges as an essential indicator of hydration status, with implications for AIS prognosis [19, 20]. A previous study has revealed a positive correlation between reduced BCR and early neurological improvement in patients with AIS [21]. However, the effect of early BCR correction via enhanced hydration on the efficacy of thrombolytic treatments such as rt-PA should be further investigated.

## 2. Methods

This single-arm interventional study was conducted at Chia-Yi Chang Gung Memorial Hospital from May 2021 to October 2023. A historical control group was included for comparison. The historical control group enrolled consecutive patients who met the inclusion and exclusion criteria from October 2007 to December 2012. The current study primarily aimed to assess the efficacy of enhanced hydration therapy (intervention) versus standard hydration therapy (control) in patients with AIS who presented with an elevated BCR and who received rt-PA (population). The primary outcome was the rate of early neurological deterioration (END) (end point) within 72 h and improved 3-month functional outcomes (outcome).

### 2.1. Ethics Statement

The Institutional Review Board of Chia-Yi Chang Gung Memorial Hospital approved the current study on September 21, 2020 (approval number: 202001385A3). A written informed consent was obtained from all participants.

### 2.2. Study Population and Eligibility Criteria

The inclusion criteria were patients who received fibrinolytic therapy with rt-PA and had a BCR of > 15 upon admission, those with controlled blood pressure (systolic blood pressure < 185 mmHg and diastolic blood pressure < 110 mmHg), and those without major contraindications, including recent major surgery, active internal bleeding, and anticoagulant use. rt-PA therapy was indicated for patients with a confirmed diagnosis of AIS, as validated via neuroimaging (e.g., computed tomography scan and magnetic resonance imaging), who had a therapeutic window of up to 4.5 h from symptom onset and a National Institutes of Health Stroke Scale (NIHSS) score of 4–25.

The exclusion criteria were as follows: patients with heart failure with a left ventricular ejection fraction < 45%, chronic renal disease with an estimated glomerular filtration rate < 50 mL/min/1.73 m^2^, those receiving active cancer treatment and diuretics, those with an initial systolic blood pressure < 90 mmHg upon emergency department arrival, those with fever (core temperature: ≥ 38°C), those with medical conditions requiring aggressive hydration or blood transfusions, those who cannot complete the 3-month follow-up, those who refused to participate, and those with low blood pressure due to the need for significantly high fluid volumes, which could result in deviations from the study protocol.

### 2.3. Management of Patients Who Were Enrolled and Study Interventions

After rt-PA administration, patients in the enhanced hydration therapy group received 0.9% NaCl intravenous infusion at a volume of 20 mL/kg body weight. A one-third bolus dose was administered initially. Then, the remaining volume was gradually infused over 8 h. Intravenous 0.9% NaCl was continuously infused at a rate of 40–80 mL/h for an additional 16 h. All infusions were precisely controlled with infusion pumps to ensure accurate dosing. In contrast, the historical control group received normal saline at a rate of 40–60 mL/h within the first 24 h.

### 2.4. Diagnostic Assessments

Data including age, sex, time of symptom onset, initial signs and symptoms, medical history, smoking status, electrocardiogram results, arterial blood pressure upon emergency department admission, and blood biochemistry results were collected using a standardized form in the emergency department. Stroke severity upon admission and after 72 h was assessed using the NIHSS. Additional data such as length of hospital stay and modified Rankin Scale (mRS) scores at 3 months were obtained.

### 2.5. Stroke Severity Assessments and Study Outcomes

Stroke severity was evaluated using the NIHSS, and functional outcomes were investigated using the mRS [22, 23]. Neurologists who were blinded to the study examined the patients upon admission, within the first 3 days of hospitalization, and prior to discharge. END was defined as an increase in the NIHSS score of ≥ 4 within 72 h. A favorable functional outcome was defined as an mRS score of 2 at ≤ 3 months after stroke.

### 2.6. Statistical Analysis

Continuous variables were expressed as mean ± standard deviation and assessed for normality using the Shapiro–Wilk test. Based on distribution, data were compared using either the two-sample *t* test for data with a normal distribution or the Mann–Whitney *U* test for data with a non-normal distribution. Categorical data were expressed as numbers (percentages) and compared using the Pearson chi-square test. All statistical analyses were performed using the Statistical Package for the Social Sciences software Version 29. A *p* value of < 0.05 indicated statistically significant differences.

## 3. Results

In total, 92 patients who received rt-PA treatment were initially screened. Among them, patients with a BCR < 15 (*n* = 67); those with heart failure (*n* = 2), an initial systolic blood pressure < 90 mmHg (*n* = 1), and an estimated glomerular filtration rate < 50 mL/min/1.73 m^2^ (*n* = 1); and those who declined to participate and did not provide informed consent (*n* = 1) were excluded. Consequently, 20 participants were enrolled in the enhanced hydration group and 170 in the historical control group.

As shown in Table 1, the enhanced hydration and historical control groups did not significantly differ in terms of sex distribution, age, systolic blood pressure, and body mass index. However, the enhanced hydration group had a higher incidence of hypertension than the historical control group (85% vs. 60%, *p*=0.03). There were no significant differences in the prevalence of diabetes, hyperlipidemia, coronary artery disease, atrial fibrillation, stroke history, or chronic kidney disease. The enhanced hydration group had a slightly lower NIHSS score upon admission than the historical control group (10.2 ± 5.5 vs. 12.5 ± 5.7, *p*=0.09). The two groups were similar in terms of premorbid functional status and laboratory findings.

As shown in Table 2, in terms of outcomes, the enhanced hydration group had a significantly higher rate of favorable functional outcomes (mRS score ≤ 2 at 3 months) than the historical control group. In particular, 45% of the participants in the enhanced hydration group and 21.2% in the control group achieved favorable outcomes (*p*=0.018). There was no significant difference in the incidence rate of END between the enhanced and standard hydration groups (10.0% vs. 10.6%, *p*=0.661).

In a subgroup analysis based on stroke severity, in the enhanced hydration group, patients with a low stroke severity (NIHSS score of 4–10) had significantly better 3-month outcomes than those with a high stroke severity (NIHSS score of 11–24). In particular, 75% of patients in the enhanced hydration group and 31.5% in the control group achieved favorable outcomes (*p*=0.004). However, the enhanced hydration and historical control groups did not significantly differ in the proportion of patients with a higher stroke severity who had favorable outcomes (0% vs. 13.4%, *p*=0.269).

Based on an analysis of stroke subtypes using the Trial of ORG 10172 in Acute Stroke Treatment (TOAST) criteria, the functional outcomes between the enhanced hydration and historical control groups did not significantly differ. However, after excluding patients who underwent endovascular thrombectomy (EVT), the enhanced hydration group had a higher rate of 3-month favorable outcomes than the historical control group (56.3% vs. 21.2%, *p*=0.002).

Regarding complications, the enhanced hydration group was more likely to present with hemorrhagic transformation than the historical control group (20% vs. 10%, *p*=0.177). However, the results did not significantly differ. Further, the two groups did not significantly differ in terms of the incidence of brain swelling requiring craniectomy or congestive heart failure. In a subgroup analysis, patients with a low stroke severity did not present with significant complication rates (Table 3).

Finally, none of the patients in the enhanced hydration group developed adverse events such as edema, rigor, fever, dyspnea, and congestive heart failure.

## 4. Discussion

This preliminary study emphasized the potential impact of enhanced hydration on the prognosis of patients with AIS who had an elevated BCR and who received rt-PA treatment. Our analysis showed that the study group presented with a significant improvement in functional outcomes, as indicated by mRS scores of ≤ 2 at 3 months, compared with the historical control group. Based on this finding, correcting hypovolemia might improve the prognosis of patients with AIS who received rt-PA treatment.

Hypercatecholemia and other factors can contribute to an elevated BCR [24]. However, in majority of cases, a high BCR is likely attributed to hypovolemia. In such cases, enhanced hydration can be significantly beneficial. A recent clinical trial supports this viewpoint. In particular, patients with AIS who had an elevated BCR and who received enhanced hydration had improved outcomes compared with those with minor stroke and nonlarge vessel occlusion [25].

When stratified according to baseline NIHSS scores, enhanced hydration was significantly beneficial for patients with a low stroke severity. However, it had no significant benefit in patients with a high stroke severity. This disparity may be due to the possibility that enhanced hydration can exacerbate brain edema in patients with high stroke severity, thereby potentially diminishing the benefits of correcting hypovolemia [26, 27].

In addition, in our study, the study and historical control groups did not significantly differ in terms of the incidence of END. Thus, the benefits of enhanced hydration may be more related to facilitating recanalization, leading to better long-term outcomes, rather than preventing neurological deterioration if recanalization is not achieved [28]. However, this hypothesis should be further validated.

Considering the efficacy of EVT in AIS treatment, additional analyses of patients who did not receive EVT was conducted to further assess the impact of correcting hypovolemia on recanalization. Results showed that the study group exhibited a significant improvement in achieving 3-month favorable outcomes, thereby highlighting the importance of addressing hypovolemia in this subset of patients [29–31].

The lack of significant differences in favorable outcomes across stroke subtypes based on the TOAST criteria at 3 months is an interesting area of further research. Previous studies have revealed that patients with lacunar infarctions may benefit from enhanced hydration, leading to improved outcomes over a 3-month period [32]. However, the small sample size in our study might have limited the statistical power of these findings, which underscored the need for large-scale randomized clinical trials.

The study and historical control groups did not significantly differ in terms of complication rates. However, patients with a low stroke severity in the study group had a lower rate of hemorrhagic transformation, which decreased from 20% to 8.3%. These preliminary results indicate the efficacy and safety of enhanced hydration, thereby suggesting that further comprehensive trials should focus on patients with AIS who have a low stroke severity and who are receiving thrombolytic therapy. Nevertheless, future studies should explore the timing, dosage, and method of hydration therapy combined with rt-PA administration to optimize AIS management.

To the best of our knowledge, this study first showed the potential beneficial effect of enhanced hydration in patients receiving thrombolytic treatment. Our findings indicate that patients with a low stroke severity may have a greater benefit from this intervention. The findings of the current study are in accordance with those of a recent clinical trial, which further supports the potential role of a high BCR in guiding treatment decision-makings for patients with AIS [25].

This study has several limitations that should be acknowledged. First, it has a small sample size (*n* = 20), which limited the generalizability of the findings and reduced the statistical power to detect subtle effects. While the results demonstrate statistical significance, caution should be exercised in interpreting these findings, given the limited sample size, which may not fully represent broader populations. Second, the inclusion of a historical control group, rather than the use of a randomized controlled trial design, introduced potential confounding factors that may have influenced the results. Finally, as this is a pilot study, the findings should be considered preliminary. They are intended to provide a foundation for hypothesis generation and inform the design of future research.

## 5. Conclusion

Enhanced hydration is associated with better 3-month functional outcomes in patients with AIS who are receiving thrombolytic treatment. However, this finding is preliminary. Thus, further investigations using a randomized controlled trial design should be conducted to validate the efficacy of such an intervention in routine clinical practice.

## Figures and Tables

**Table 1:** Clinical profiles of the two groups.

Characteristics of the participants	Study group (*n* = 20)	Historical control group (*n* = 170)	*p* value
*Sex* ^2^			
Female	6 (30.0%)	77 (45.3%)	0.19

Age, years^1^	66.3 ± 10.0	66.2 ± 12.2	0.84

BMI^1^	24.1 ± 2.9	24.0 ± 3.6	0.83

Systolic blood pressure, mmHg^1^	156.7 ± 32.2	144.9 ± 21.8	0.06

*Medical*⁣*history*^2^			
Hypertension	17 (85.0%)	102 (60.0%)	0.03^∗^
Diabetes mellitus	8 (40.0%)	50 (35.3%)	0.33
Hyperlipidemia	6 (30.0%)	55 (32.4%)	0.83
Coronary artery disease	2 (10.0%)	6 (3.5%)	0.17
Atrial fibrillation	7 (35.0%)	56 (32.9%)	0.85
Previous stroke (including TIA)	3 (15.0%)	31 (18.2%)	0.72
Chronic kidney disease	0 (0%)	3 (1.8%)	0.55

Baseline NIHSS score^1^	10.2 ± 5.5	12.5 ± 5.7	0.09

*Premorbid*⁣*mRS*⁣*score*^2^			
0–2	20 (100%)	156 (91.8%)	0.35

*Laboratory*⁣*tests*^1^			
WBC count (10^3^/µL)	9.4 ± 3.0	8.2 ± 3.3	0.06
Hemoglobin level (g/dL)	14.3 ± 1.6	13.9 ± 1.8	0.18
Platelet count (10^3^/µL)	219.9 ± 69.5	213.3 ± 61.3	0.72
Glucose level upon ER admission (mg/dL)	140.5 ± 44.9	153.1 ± 66.3	0.78
ALT level (U/L)	24.7 ± 9.5	28.0 ± 16.2	0.69
BUN level (mg/dL)	17.9 ± 3.7	19.2 ± 8.9	0.81
Creatinine level (mg/dL)	0.9 ± 0.2	0.9 ± 0.3	0.66
BCR upon admission	20.3 ± 3.5	21.3 ± 9.3	0.79
Total cholesterol level (mg/dL)	178.7 ± 43.9	180.7 ± 39.8	0.91
Triglyceride level (mg/dL)	98.2 ± 31.2	115.9 ± 65.2	0.55
Uric acid level (mg/dL)	5.4 ± 1.8	5.4 ± 1.7	0.91
Prothrombin time (s)	10.7 ± 0.6	11.1 ± 1.2	0.06
APTT (s)	26.4 ± 2.9	26.1 ± 2.8	0.31

*Note:* ALT, alanine aminotransferase; BCR, blood urea nitrogen-to-creatinine ratio; HbA1c, hemoglobin A1c.

Abbreviations: APTT, activated partial thromboplastin time; BMI, body mass index; BUN, blood urea nitrogen; CAD, coronary artery disease; LDL, low-density lipoprotein; NIHSS, National Institutes of Health Stroke Scale; WBC, white blood cell.

^1^Continuous data were expressed as mean ± SD.

^2^Categorical data as number (%).

^∗^ *p* < 0.05 indicated statistically significant difference.

**Table 2:** Outcomes between the study and historical control groups.

**Variables**	**Study group (*n* = 20)**	**Historical control group (*n* = 170)**	**p** **value**

mRS scores ≤ 2 at 3 months	9 (45.0%)	36 (21.2%)	0.018^∗^
END (increase in the NIHSS score of ≥ 4) within 72 h	2 (10.0%)	18 (10.6%)	0.661

**Subgroup analysis of mRS scores ≤ 2 at 3 months**	**Study group**	**Historical control group**	**p** **value**

Baseline NIHSS score of 4–10 (%, *n*)	9 (75%, 12)	23 (31.5%, 73)	0.004^∗^
Baseline NIHSS score of 11–24 (%, *n*)	0 (0%, 8)	13 (13.4%, 97)	0.269
Lacunar infarction^a^ (%, *n*)	3 (60%, 5)	12 (38.7%, 31)	0.370
Large-artery atherosclerosis^a^ (%, *n*)	3 (37.5%, 8)	7 (17.9%, 39)	0.218
Cardioembolism^a^ (%, *n*)	1 (8.3%, 12)	5 (7.8%, 64)	0.945
No history of EVT (%, *n*)	9 (56.3%, 16)	36 (21.2%, 170)	0.002^∗^

*Note:* Data were presented as *n* (%) unless otherwise stated.

Abbreviations: END, early neurological deterioration; EVT, endovascular thrombectomy; mRS, modified Rankin scale; NIHSS, National Institutes of Health Stroke Scale.

^a^Classified according to the “Trial of ORG 10,172 in Acute Stroke Treatment” classification.

^∗^ *p* < 0.05.

**Table 3:** Complications between the study and historical control groups.

**Variables**	**Study group (*n* = 20)**	**Historical control group (*n* = 170)**	**p** **value**

Hemorrhagic transformation	4 (20.0%)	17 (10.0%)	0.177
Brain swelling requiring craniectomy	1 (5.0%)	4 (2.4%)	0.484
Congestive heart failure	0 (0%)	2 (1.2%)	0.626

**Subgroup analysis of low stroke severity (NIHSS score of 4–10)**	**Study group (*n* = 12)**	**Historical control group (*n* = 73)**	**p** **value**

Hemorrhagic transformation	1 (8.3%)	6 (8.2%)	0.989
Brain swelling requiring craniectomy	0 (0%)	1 (1.4%)	0.683
Congestive heart failure	0 (0%)	0 (0%)	

*Note:* Data were presented as *n* (%) unless otherwise stated.

Abbreviation: NIHSS, National Institutes of Health Stroke Scale.

^∗^ *p* < 0.05.

## Data Availability

The data that support the findings of this study are available from the corresponding author upon reasonable request.
